# Elevation of ambient temperature is associated with an increased risk of herpes zoster: a time-series analysis

**DOI:** 10.1038/s41598-019-48673-5

**Published:** 2019-08-22

**Authors:** Yoon-Jung Choi, Youn-Hee Lim, Kyung-Shin Lee, Yun-Chul Hong

**Affiliations:** 1 Department of Preventive Medicine, Seoul National University College of Medicine, Seoul, Republic of Korea; 2 Institute of Environmental Medicine, Seoul National University Medical Research Center, Seoul, Republic of Korea; 3 Environmental Health Center, Seoul National University College of Medicine, Seoul, Republic of Korea

**Keywords:** Environmental health, Epidemiology

## Abstract

Although varicella zoster (VZ) and herpes zoster (HZ) are caused by the same varicella zoster virus (VZV), the former is caused by primary infection while the latter is caused by reactivation of latent VZV, and their relationships with ambient temperature are also different. It is relatively well-established that VZ incidence declines with ambient temperature, but the relationship between HZ and ambient temperature is inconclusive. Thus, we investigated the effects of ambient temperature on the incidence of HZ in time-series analysis by using data from the Korean National Emergency Department Information System between 2014 and 2016. We applied a generalized linear model to investigate the relationship between ambient temperature and emergency room (ER) visits due to HZ, after controlling for confounders in seven metropolitan cities and nine provinces in South Korea. Region-specific estimates were pooled to obtain the national average estimates. There were a total of 61,957 ER visits nationwide for HZ during the study period. HZ significantly increased by 2.03% to 2.94% in the moving average lag models throughout 0 to 11 days with maximum percent increase of 2.94% (95% CI: 2.20, 3.68) in the 6-day moving average lag model.

## Introduction

Reactivation of latent varicella zoster virus (VZV) infection can cause herpes zoster (HZ) (shingles) decades after the primary VZV infection in childhood, which is known as varicella zoster (VZ) (chicken pox). Although HZ mostly resolves spontaneously, neurologic complications can develop, including post-herpetic neuralgia, cranial nerve palsies, myelitis, and encephalitis. The lifetime risk of HZ has been reported to be as high as 32% in Taiwan and 30% in the United Kingdom^1,2^. Severity also increases after the age of 50 years^2^.

VZ and HZ are caused by the same virus, but the mechanisms and clinical entities are different; the former is caused by airborne transmission as well as direct contact of VZV, mostly in children or younger ages, and the latter is caused by the reactivation of latent VZV in older adults, especially when accompanied by risk factors such as immunosuppressive conditions and chronic diseases including heart failure, COPD, asthma, chronic kidney disease, and depression^3–5^. The effects of meteorological factors on VZ and HZ are also different. VZV is not well transmitted under conditions of high ambient temperatures and high humidity^6^. The seroprevalence of VZV in Europe is the lowest in the Mediterranean region where the temperatures are the highest in Europe^7,8^. The reported VZ incidence is higher during the cooler seasons in South and Southeast Asia^6,9,10^, and ambient temperature is negatively associated with VZ incidence in Greece^11^ and Shanghai, China^12^.

In contrast, HZ incidence has been reported to be positively associated with increased ambient temperature. Although some previous studies failed to detect seasonality of HZ^1,13–15^, which could be due to (1) using admission or consultation data which is more likely to involve HZ complications which are more chronic in nature or (2) higher latitudes of the regions in which HZ seasonality was not observed, studies in Japan and Taiwan showed that the prevalence of HZ was high in summer and low in winter, which is a mirror image of VZ incidence^16,17^. However, these studies did not quantify the degree of association between ambient temperature and HZ. A time-series analysis in Australia reported that HZ was positively associated with ambient temperature and UV light; however, the study was limited to a single city, and there were a total of 3,690 HZ cases over 5 years^18^. Another time-series analysis in China showed that a 1 °C increase in daily mean temperature was associated with a 2.18% increase in outpatient dermatology visits due to HZ; however, the study was confined to a single medical center in Shanghai, with a total of 6,614 HZ cases^12^.

Although the association between ambient temperature and VZ is relatively well established, evidence regarding the association between ambient temperature and HZ is not convincing. Limited sample sizes in previous studies indicate a need for more comprehensive data analysis over greater geographic areas. Thus, we used large-scale, nationwide, emergency department data to confirm the association between meteorological factors and HZ, focusing on ambient temperature. This enabled us to further analyze the differential effects of temperature by sex and age groups.

## Results

**Figure 1:**
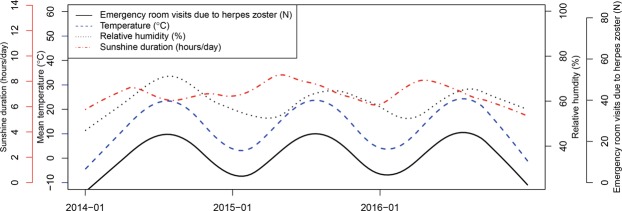
Mean temperature, total sunshine duration, and emergency room visits for herpes zoster in Seoul, Korea (2014–2016).

There were a total of 61,957 ER visits due to HZ without complications nationwide in South Korea from January 1, 2014 to December 31, 2016, with 25,355 and 36,602 male and female patients, respectively. According to age groups, there were 2,148 patients with HZ aged 0–18 years, 42,427 aged 19–64 years, and 17,382 aged ≥65 years. Over the given study period for all cities and provinces, mean temperature, relative humidity, and sunshine duration were 13.80 ± 9.30 °C, 68.70 ± 15.34%, and 6.28 ± 3.87 hours/day, respectively (Table 1). Seasonal variation of HZ incidence coincided with seasonal variation of mean temperature in Seoul (Fig. 1). However, the seasonal pattern of relative humidity and sunshine duration did not quite overlap with seasonal variation of HZ incidence. When confined to Seoul, the Pearson correlation coefficients between ER visits for HZ and mean temperature, relative humidity, and sunshine duration were 0.14, 0.036, and −0.056, respectively.

**Table 1:** Descriptive statistics for meteorological data and cases of herpes zoster in the Republic of Korea from January 1, 2014 to December 31, 2016.

Variables	Mean ± SD	Minimum	Maximum
**Meteorological measures**			
Mean temperature (°C)	13.80 ± 9.30	−14.4	32.4
Minimum temperature (°C)	9.43 ± 9.69	−18.12	28.7
Maximum temperature (°C)	18.92 ± 9.43	−10.5	38.3
Relative humidity (%)	68.70 ± 15.34	20.6	99.9
Sunshine duration (hours/day)	6.28 ± 3.87	0	13.9
**Daily emergency room visits due to herpes zoster**			
Male sex	1.45 ± 2.61	0	39
Female sex	2.09 ± 3.71	0	55
Age 0–18 years	0.12 ± 0.40	0	6
Age 19–64 years	2.42 ± 4.39	0	67
Age ≥65 years	0.99 ± 1.77	0	26
Total	3.53 ± 6.03	0	91

**Figure 2:**
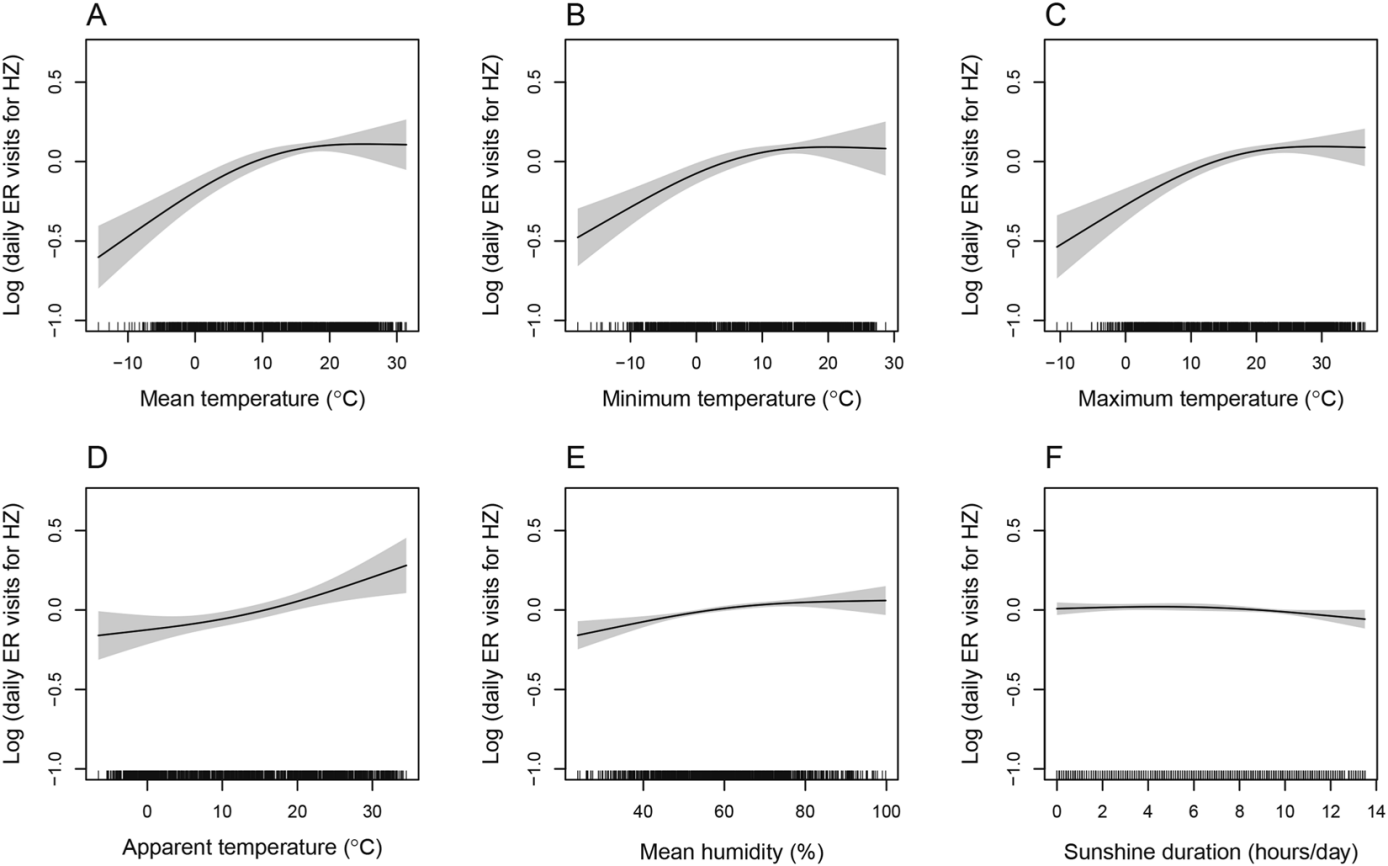
Generalized additive model (GAM) showing association between the log of daily number of visits for herpes zoster in emergency departments in Seoul, Korea and (**A**) mean temperature, (**B**) minimum temperature, (**C**) maximum temperature, (**D**) apparent temperature, (**E**) relative humidity, and (**F**) sunshine duration (Abbreviation: ER, emergency room; HZ, herpes zoster).

As shown in Fig. 2, the generalized additive model (GAM) analysis indicated a positive association between HZ and mean temperature in Seoul when adjusted for relative humidity, duration of sunshine, and days of the week. Minimum, maximum, and apparent temperature documented for Seoul also had positive associations with HZ while the associations between HZ and relative humidity or sunshine duration were not significant in Seoul (Fig. 2). Other cities and provinces showed similar positive associations between HZ and mean temperature (Supplementary Fig. 1).

**Figure 3:**
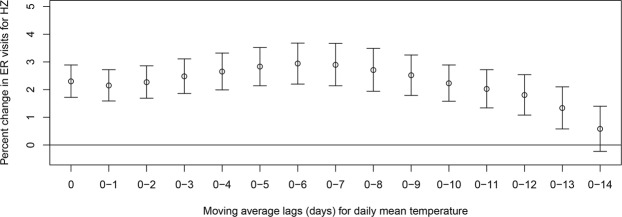
Pooled estimates of percent change in emergency room visits for herpes zoster with every 1 °C increase in mean temperature in up to 14-day moving average lag models, from the meta-analysis of a generalized linear model (GLM) for seven metropolitan cities and nine provinces in Korea (Abbreviation: ER, emergency room; HZ, herpes zoster).

Table 2 and Fig. 3 show the pooled estimates resulting from meta-analysis of the generalized linear model (GLM) estimates for each city and province in the models of up to 14-day moving average lags (the results of meta-analysis for each city and province are shown in Supplementary Fig. 2). In the 1-day lag model, the percent change in ER visits for HZ according to a 1-unit increase in ambient temperature (°C) was 2.15% (95% confidence interval (CI): 1.59, 2.72). In the 7-day moving average lag model, ER visits for HZ increased by 2.90% (95% CI: 2.14, 3.67) with every 1 °C increase in daily mean temperature. As lag days increased in the moving average models, percent changes of HZ with increases in ambient temperature remained above 2% (2.03–2.94%), up to the 11-day moving average lag models, followed by a decrease in percent changes of HZ thereafter, reaching a nonsignificant level. The maximum percent increase of HZ cases was 2.94% (95% CI: 2.20, 3.68) by 1 °C increase in daily mean temperature in the 6-day moving average lag model.

**Table 2:** Pooled estimates of percent change in emergency room visits due to herpes zoster according to a 1 °C increase in daily mean temperature using different moving average lag models from January 1, 2014 to December 31, 2016 in the meta-analysis of a generalized linear model (GLM) for seven metropolitan cities and nine provinces in Korea.

Moving average lag	Percent change (95% CI)
0 lag	2.30 (1.72, 2.89)
0–1	2.15 (1.59, 2.72)
0–2	2.27 (1.69, 2.86)
0–3	2.48 (1.86, 3.11)
0–4	2.65 (1.99, 3.32)
0–5	2.83 (2.14, 3.52)
0–6	2.94 (2.20, 3.68)
0–7	2.90 (2.14, 3.67)
0–8	2.71 (1.94, 3.49)
0–9	2.52 (1.79, 3.25)
0–10	2.23 (1.58, 2.89)
0–11	2.03 (1.34, 2.72)
0–12	1.81 (1.08, 2.54)
0–13	1.34 (0.58, 2.10)
0–14	0.58 (−0.23, 1.40)

Abbreviation: CI, confidence interval.

**Figure 4:**
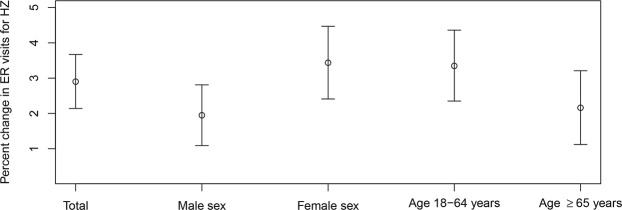
Pooled estimates of percent change in emergency room visits due to herpes zoster with every 1 °C increase of mean temperature in the 7-day moving average lag model by sex and age group from the meta-analysis of a generalized linear model (GLM) for seven metropolitan cities and nine provinces in Korea (Abbreviation: ER, emergency room; HZ, herpes zoster).

When classified by sex and age subgroups, a 1 °C increase in daily mean temperature was associated with percent changes in HZ of 2.12% (95% CI: 1.39, 2.85) for male patients, 2.28% (95% CI: 1.68, 2.89) for female patients, 2.38% (95% CI: 1.70, 3.05) for patients aged 19–64 years, and 2.13% (95% CI: 1.30, 2.97) for those ≥65 years of age in the 0-lag model (Table 3 and Fig. 4). In the 7-day moving average model, percent changes of HZ were 1.95% (95% CI: 1.09, 2.81) in male patients, 3.44% (95% CI: 2.41, 4.47) in female patients, 3.35% (95% CI: 2.35, 4.36) in patients aged 19–64 years, and 2.16% (95% CI: 1.12, 3.21) in those ≥65 years of age. These results indicated greater effect sizes in female than in male patients and in patients aged 19–64 years than those aged ≥65 years; however, the differences of effect sizes between the groups were not statistically significant.

**Table 3:** Pooled estimates of percent change in emergency room visits due to herpes zoster according to a 1 °C increase in daily mean temperature by sex and age groups, using different moving average lag models from January 1, 2014 to December 31, 2016 from the meta-analysis of a generalized linear model (GLM) for seven metropolitan cities and nine provinces in Korea.

Moving average lag (days)	Total	Male sex	Female sex	Age 19–64 years	Age ≥65 years
0	2.3 (1.72, 2.89)	2.12 (1.39, 2.85)	2.28 (1.68, 2.89)	2.38 (1.70, 3.05)	2.13 (1.30, 2.97)
0–1	2.15 (1.59, 2.72)	2.00 (1.28, 2.73)	2.13 (1.49, 2.77)	2.30 (1.62, 2.98)	1.90 (1.11, 2.69)
0–7	2.90 (2.14, 3.67)	1.95 (1.09, 2.81)	3.44 (2.41, 4.47)	3.35 (2.35, 4.36)	2.16 (1.12, 3.21)
0–14	0.58 (−0.23, 1.40)	−0.52 (−1.99, 0.98)	1.23 (0.18, 2.28)	0.77 (−0.22, 1.76)	0.43 (−1.06, 1.94)

## Discussion

We studied the relationship between daily mean temperature and ER visits for HZ using a time-series analysis. Meta-analysis of seven metropolitan cities and nine provinces of South Korea revealed that for every 1 °C increase in ambient temperature, HZ significantly increased by 2.03% to 2.94% in the moving average lag models from 0 to 11 days with maximum percent increase of 2.94% (95% CI: 2.20, 3.68) in the 6-day moving average lag model. Previous studies have investigated the association between HZ and meteorological factors, but these studies were confined to a single medical center or a single city, and the number of cases of HZ was limited. To the best of our knowledge, this is the first large-scale time-series analysis of the association between ER visits for HZ and ambient temperature covering the entire nation of South Korea. The use of large-scale data enabled subgroup analysis by sex and age groups, with percent changes found to be greater in female patients and patients aged 18–64 years, although the difference was not statistically significant.

Little is known about the underlying mechanisms of seasonal variations in HZ incidence. VZV-specific T-cell immunity is acquired during primary infection, which is essential for host recovery from VZ^19^. In the later life of a host, VZV reactivates and reaches the skin via axonal transport, presenting the characteristic skin lesions along the dermatome. Memory T cells that recognize VZV proteins remain detectable after primary infection, and VZV reactivation becomes more frequent as the interval from the primary infection lengthens^19^. Previous studies have shown that a decline in T-cell recognition of VZV antigens is associated with increased incidence of HZ^20^. In fact, HZ reactivation is known to be associated with a spectrum of immunosuppression such as immunosenescence which refers to the natural decline in T-cell function as aging, disease-induced immunosuppression, and immunosuppressive therapy^21^.

There is some evidence that host immune function could also be affected by environmental factors such as seasons and ambient temperature. Seasonality of infectious diseases are well-established in humans, and it has been suggested that inborn immunological rhythm underlies infectious diseases seasonality^22^. If seasonality of infectious diseases is related with seasonality of host immune system, then enhanced or reduced immune response would, in turn, affect reactivation of latent viral infection. Previous studies have shown that human innate and adaptive immune responses show variability according to seasons. In summer, a decrease is seen in the percentage of monocytes expressing toll-like receptors (TLRs) and pro-inflammatory cytokines, such as interleukin (IL)-1β, IL-6, tissue necrosis factor (TNF), IL-10, and IFN-γ. Circulating CD4+ and CD8+ T cell levels increase and anti-inflammatory regulatory T cell levels decrease in summer^23,24^. The mRNA expression of genes involved in B-cell receptor signaling, chemokine signaling, and phagosome were increased in winter and decreased in summer^25^. This pattern was linearly predicted by daily mean ambient temperature and sunlight duration, and was not observed in people residing in an equatorial regions. Dopico *et al*. suggested that seasonal gene expression was evolutionarily selected for such that immune response may be enhanced as a result of co-evolution with infectious microorganisms and more intense inter-species competition during winter^25^. Thus, it is probable that relatively reduced level of immune response in summer leaves people more susceptible to reactivation of latent viral infection.

Along with temperature, ultraviolet radiation (UVR) and vitamin D may also contribute to the seasonality of HZ. In humans, UVR-mediated immunosuppression is associated with reactivation of latent infection of herpes simplex virus^26^, which belongs to the same Alphaherpesvirinae subfamily as VZV. Exposure to UVR is associated with downregulation of microorganism-specific T cells and decreased production of antibodies by B-cells^27^. In contrast, vitamin D is widely known for its roles in immune enhancement^28^. Recent studies identified the association between lower serum vitamin D levels and increased rates of infectious diseases such as Tb^29,30^, acute respiratory tract infection^31^, and malaria^32^. These associations were greater at higher or lower latitudes where vitamin D deficiency is greater compared to equatorial regions^28^. The sunshine duration is associated with UVR and vitamin D as well as ambient temperature; therefore, further studies using direct information of UVR and vitamin D are required to confirm the effects of ambient temperature on HZ.

The main limitation of this study is that the data only included ER visits; thus, the total number of HZ cases, including those in outpatient clinics, is unknown. Nonetheless, the use of national ER data is reasonable for detection of trends in HZ incidence according to ambient temperature changes. Another limitation is that although we excluded HZ cases with complications, it is possible that the ICD-10 code was misclassified. For instance, the ICD-10 code for HZ without complications might have been assigned to patients with HZ who had complications and vice versa. Inclusion of patients with HZ who had complications could result in misleading effects of ambient temperature on the incidence of HZ because complications could be present in a more chronic fashion, such as post-herpetic neuralgia.

In conclusion, our study was the first large-scale time-series analysis that showed a significant association of elevation of ambient temperature with increased HZ incidence. While the association between ambient temperature and HZ was positive over the continuous three-year period, it would be important to conduct a study over a longer period of time, especially to find out the effects of heat waves on HZ.

## Materials and Methods

### Data of HZ and meteorological parameters

We obtained data from the National Emergency Department Information System (NEDIS) of Korea from January 1, 2014 to December 31, 2016. Cases of HZ were identified as ICD-10 code B02.9. Since we were more interested in the effect of ambient temperature on the incidence of HZ rather than sequelae of HZ, inclusion of HZ cases with complications would make the interpretation more complex. Also, HZ-related complications such as post-herpetic neuralgia could present in a more chronic fashion, leading to recurrent emergency room (ER) visits. Thus, we excluded cases of HZ with complications, such as zoster encephalitis (B02.0), zoster meningitis (B02.1), zoster with other nervous system involvement (B02.2), zoster ocular disease (B02.3), disseminated zoster (B02.7), and zoster with other complications (B02.8). We included only the main diagnosis codes rather than secondary or tertiary diagnosis codes, to exclude chronic conditions related to HZ.

Data of meteorological parameters including daily maximum, mean, and minimum temperature (°C), relative humidity (%), and sunshine duration (hours/day) were obtained from the Korea Meteorological Administration for the given study period. The weather data were extracted for seven metropolitan cities (Seoul, Busan, Daegu, Incheon, Gwangju, Daejeon, and Ulsan) and nine provinces (Kyung-ki, Kang-won, Chung-buk, Chung-nam, Jeon-buk, Jeon-nam, Kyung-buk, Kyung-nam, and Jeju) of South Korea, which virtually cover all over the country. As this study used de-identified publicly available data, the Seoul National University Hospital Institutional Review Board (IRB) deemed it exempt from IRB review (IRB No. H-1807-028-955).

### Statistical analysis

We used a generalized additive model (GAM) to investigate the relationship between meteorological factors such as ambient temperature, humidity, and sunshine duration as independent variables and ER visits due to HZ as a dependent variable with Poisson distribution. The analysis was conducted separately for each city and province. In the analysis for the effect of ambient temperature, we adjusted for mean relative humidity and sunshine duration on the same day. Day of the week was also controlled for in the model to detect distinct patterns of ER visits according to days, such as weekend or holidays. Eight degrees of freedom (*df*) per year were chosen for the natural cubic spline function of calendar time based on the lowest unbiased risk estimator (UBRE)^33,34^ (Supplementary Fig. 3). We assumed 3 *df* for confounders such as ambient temperature, humidity, and sunshine duration, based on the previous study^12^. To explore the lag patterns of the effects of ambient temperature on HZ, we fitted the model for single-day lags from 0 to 14 days and moving average lags for up to 14 days.

With an assumption of linearity based on the results of the GAM analysis, we further analyzed the association between ambient temperature and HZ using a generalized linear model (GLM) separately for each city and province. Similar to GAM analysis, the GLM was adjusted for mean humidity, sunshine duration, and day of the week. We selected 8 *df* per year for the natural cubic spline function of calendar time based on Akaike Information Criterion (AIC)^35,36^ (Supplementary Fig. 4). Degrees of freedom for mean temperature, humidity, and duration of sunshine were given as identical as in the GAM analysis. Estimates from the GLM analysis for all the cities and provinces were subjected to meta-analysis to compute pooled estimates in Korea for single-day lags from 0 to 14 days and moving average lags for up to 14 days. Pooled estimates were presented as percent changes in ER visits for HZ per 1 °C increase in ambient temperature.

We conducted the GLM analysis with stratification by sex and age groups (19–64 and ≥65 years). Pooled estimates for male vs. female sex, and patients aged 19–64 vs. ≥65 years were compared using the following equation^37,38^:

$$({{\rm{\beta }}}_{1}-{{\rm{\beta }}}_{2})/\sqrt{{{\rm{SE}}}_{1}^{2}+{{\rm{SE}}}_{2}^{2}}$$

where β_1_ and β_2_ are percent change estimates, and SE_1_ and SE_2_ are standard errors for male and female sex (or age 19–64 and ≥65 years), respectively.

Statistical analyses were performed using R software (R version 3.5.1; The R Foundation for Statistical Computing, Vienna, Austria). We used the mgcv, splines, and tsModel packages in R for the GAM and GLM analyses and the metafor package for meta-analysis. Estimates with a p-value less than 0.05 were considered statistically significant.

## Supplementary information

Supplementary information

## Data Availability

The original data of meteorological variables can be found at Korea Meteorological Administration website, http://web.kma.go.kr/eng/index.jsp.

## References

[1] Brisson M (2001). Epidemiology of varicella zoster virus infection in Canada and the United Kingdom. Epidemiol Infect.

[2] Lin YH (2010). Disease burden and epidemiology of herpes zoster in pre-vaccine Taiwan. Vaccine.

[3] Forbes HJ (2014). Quantification of risk factors for herpes zoster: population based case-control study. BMJ.

[4] Wu PH (2015). A nationwide population-based cohort study to identify the correlation between heart failure and the subsequent risk of herpes zoster. BMC Infect Dis.

[5] Wung PK (2005). Herpes zoster in immunocompromised patients: incidence, timing, and risk factors. Am J Med.

[6] Garnett GP, Cox MJ, Bundy DAP, Didier JM, Catharine JS (2009). The age of infection with varicella-zoster virus in St Lucia, West Indies. Epidemiol Infect.

[7] Bonanni P (2009). Varicella vaccination in Europe - taking the practical approach. BMC Med.

[8] Katsafadou A, Ferentinos G, Constantopoulos A, Papaevangelou V (2008). The epidemiology of varicella in school-aged Greek children before the implementation of universal vaccination. Eur J Clin Microbiol Infect Dis.

[9] Lee BW (1998). Review of varicella zoster seroepidemiology in India and Southeast Asia. Trop Med Int Health.

[10] Lolekha S (2001). Effect of climatic factors and population density on varicella zoster virus epidemiology within a tropical country. Am J Trop Med Hyg.

[11] Critselis E (2012). Time trends in pediatric hospitalizations for varicella infection are associated with climatic changes: a 22-year retrospective study in a tertiary Greek referral center. PLoS One.

[12] Yang Y (2015). The effects of ambient temperature on outpatient visits for varicella and herpes zoster in Shanghai, China: a time-series study. J Am Acad Dermatol.

[13] Kim YJ, Lee CN, Lim CY, Jeon WS, Park YM (2014). Population-based study of the epidemiology of herpes zoster in Korea. J Korean Med Sci.

[14] Lin F, Hadler JL (2000). Epidemiology of primary varicella and herpes zoster hospitalizations: the pre-varicella vaccine era. J Infect Dis.

[15] Perez-Farinos N (2007). Varicella and herpes zoster in Madrid, based on the Sentinel General Practitioner Network: 1997–2004. BMC Infect Dis.

[16] Toyama N, Shiraki K, Society of the Miyazaki Prefecture, D (2009). Epidemiology of herpes zoster and its relationship to varicella in Japan: A 10-year survey of 48,388 herpes zoster cases in Miyazaki prefecture. J Med Virol.

[17] Wu PY, Wu HD, Chou TC, Sung FC (2013). Varicella vaccination alters the chronological trends of herpes zoster and varicella. PLoS One.

[18] Korostil IA, Regan DG (2016). Varicella-Zoster Virus in Perth, Western Australia: Seasonality and Reactivation. PLoS One.

[19] Abendroth A, Arvin AM (2001). Immune evasion as a pathogenic mechanism of varicella zoster virus. Semin Immunol.

[20] Levin MJ, Hayward AR (1996). The varicella vaccine. Prevention of herpes zoster. Infect Dis Clin North Am.

[21] Eshleman E, Shahzad A, Cohrs RJ (2011). Varicella zoster virus latency. Future Virol.

[22] Fisman DN (2007). Seasonality of infectious diseases. Annu Rev Public Health.

[23] Khoo AL (2011). Regulation of cytokine responses by seasonality of vitamin D status in healthy individuals. Clin Exp Immunol.

[24] Khoo AL (2012). Seasonal variation in vitamin D(3) levels is paralleled by changes in the peripheral blood human T cell compartment. PLoS One.

[25] Dopico XC (2015). Widespread seasonal gene expression reveals annual differences in human immunity and physiology. Nat Commun.

[26] Ichihashi M, Nagai H, Matsunaga K (2004). Sunlight is an important causative factor of recurrent herpes simplex. Cutis.

[27] Moyal DD, Fourtanier AM (2008). Broad-spectrum sunscreens provide better protection from solar ultraviolet-simulated radiation and natural sunlight-induced immunosuppression in human beings. J Am Acad Dermatol.

[28] Abhimanyu & Coussens, A. K (2017). The role of UV radiation and vitamin D in the seasonality and outcomes of infectious disease. Photochem Photobiol Sci.

[29] Martineau AR (2011). Reciprocal seasonal variation in vitamin D status and tuberculosis notifications in Cape Town, South Africa. Proc Natl Acad Sci USA.

[30] Sudfeld CR (2013). Vitamin D status and incidence of pulmonary tuberculosis, opportunistic infections, and wasting among HIV-infected Tanzanian adults initiating antiretroviral therapy. J Infect Dis.

[31] Charan J, Goyal JP, Saxena D, Yadav P (2012). Vitamin D for prevention of respiratory tract infections: A systematic review and meta-analysis. J Pharmacol Pharmacother.

[32] Cusick SE, Opoka RO, Lund TC, John CC, Polgreen LE (2014). Vitamin D insufficiency is common in Ugandan children and is associated with severe malaria. PLoS One.

[33] Craven P, Wahba G (1979). Smoothing noisy data with spline functions. Numer Math.

[34] Wood SN (2004). Stable and Efficient Multiple Smoothing Parameter Estimation for Generalized Additive Models. J Am Stat Assoc.

[35] Akaike H (1974). A new look at the statistical model identification. IEEE Trans Automat Contr.

[36] Chuang YH (2011). Generalized linear mixed models in time series studies of air pollution. Atmos Pollut Res.

[37] Altman DG, Bland JM (2003). Interaction revisited: the difference between two estimates. BMJ.

[38] Han C, Hong YC (2018). Adverse health effects of ferronickel manufacturing factory on local residents: An interrupted time series analysis. Environ Int.

