# Supplementary material for: Elevation of ambient temperature is associated with an increased risk of herpes zoster: a time-series analysis
Source: Sci Rep. 2019 Aug 22;9:12254. doi: 10.1038/s41598-019-48673-5 (PMC6706431; doi:10.1038/s41598-019-48673-5)

Supplementary Information

Elevation of ambient temperature is associated with an increased risk of herpes zoster: a time-series analysis

Y.-J. Choi,[1] Y.-H. Lim,[2,3*] K.-S. Lee,[2,3] Y.-C. Hong[1,2,3]

[1]Department of Preventive Medicine, Seoul National University College of Medicine, Seoul, Republic of Korea; [2]Institute of Environmental Medicine, Seoul National University Medical Research Center, Seoul, Republic of Korea; [3]Environmental Health Center, Seoul National University College of Medicine, Seoul, Republic of Korea

[*]Correspondence address: Y-H Lim, Environmental Health Center, Seoul National University College of Medicine, 103 Daehak-ro, Jongno-gu, Seoul, 03080, Republic of Korea

Telephone: 82 2 740 8394. E-mail: yhl6600@snu.ac.kr

Supplementary Figure 1. Generalized additive model (GAM) showing the association between the log of daily number of ER visits for herpes zoster and daily mean temperature in the 6-day moving average model in seven metropolitan cities (Seoul, Busan, Daegu, Incheon, Gwangju, Daejeon, and Ulsan) and nine provinces (Kyung-ki, Kang-won, Chung-buk, Chung-nam, Jeon-buk, Jeon-nam, Kyung-buk, Kyung-nam, and Jeju) of South Korea. (Abbreviation: ER, emergency room; HZ, herpes zoster)

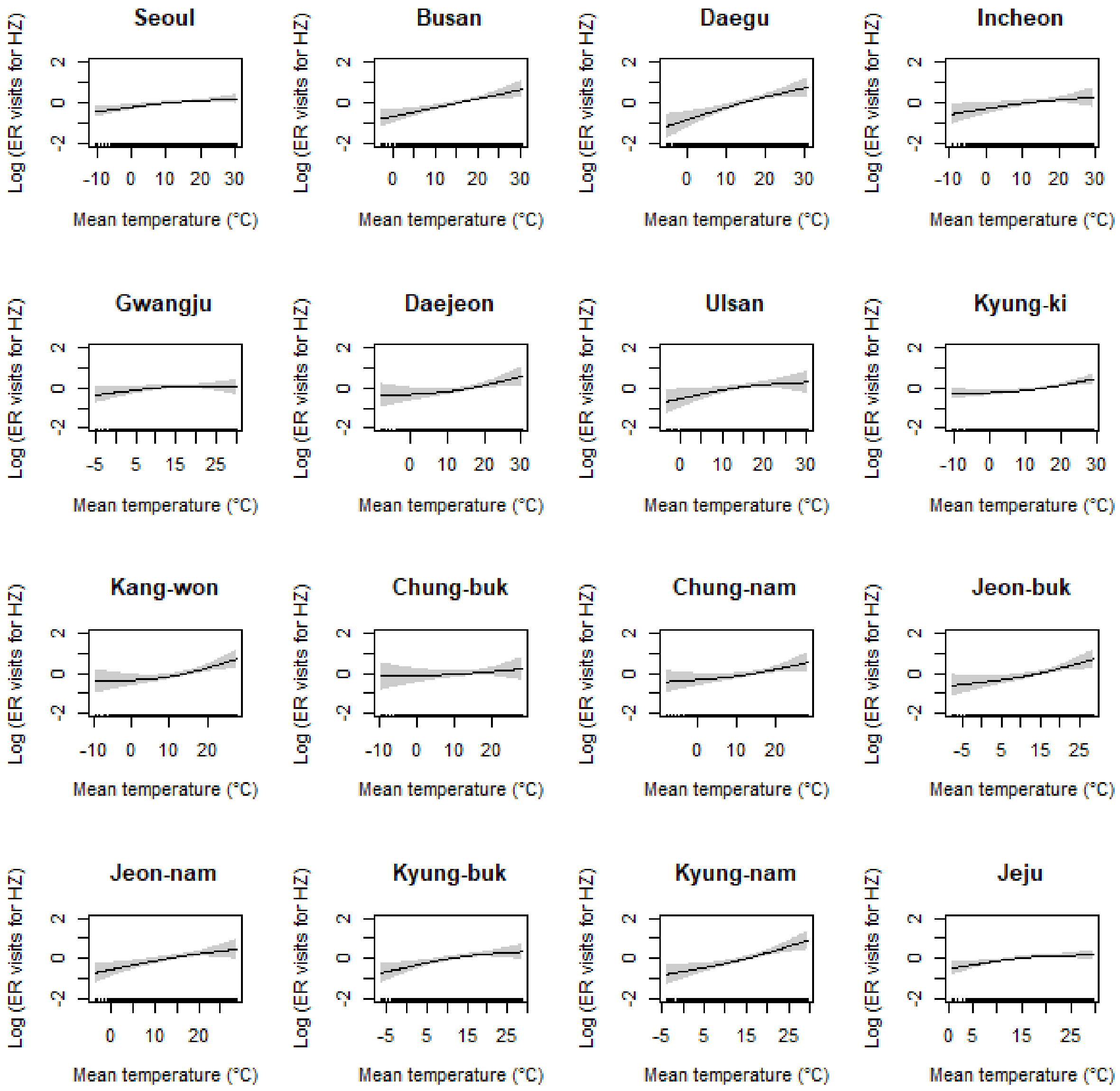

Supplementary Figure 2. Meta-analysis generalized linear model (GLM) showing percent change in ER visits due to HZ with every 1°C increase of daily mean temperature in the 6-day moving average model of seven metropolitan cities (Seoul, Busan, Daegu, Incheon, Gwangju, Daejeon, and Ulsan) and nine provinces (Kyung-ki, Kang-won, Chung-buk, Chung-nam, Jeon-buk, Jeon-nam, Kyung-buk, Kyung-nam, and Jeju) in South Korea. (Abbreviation: ER, emergency room; HZ, herpes zoster)

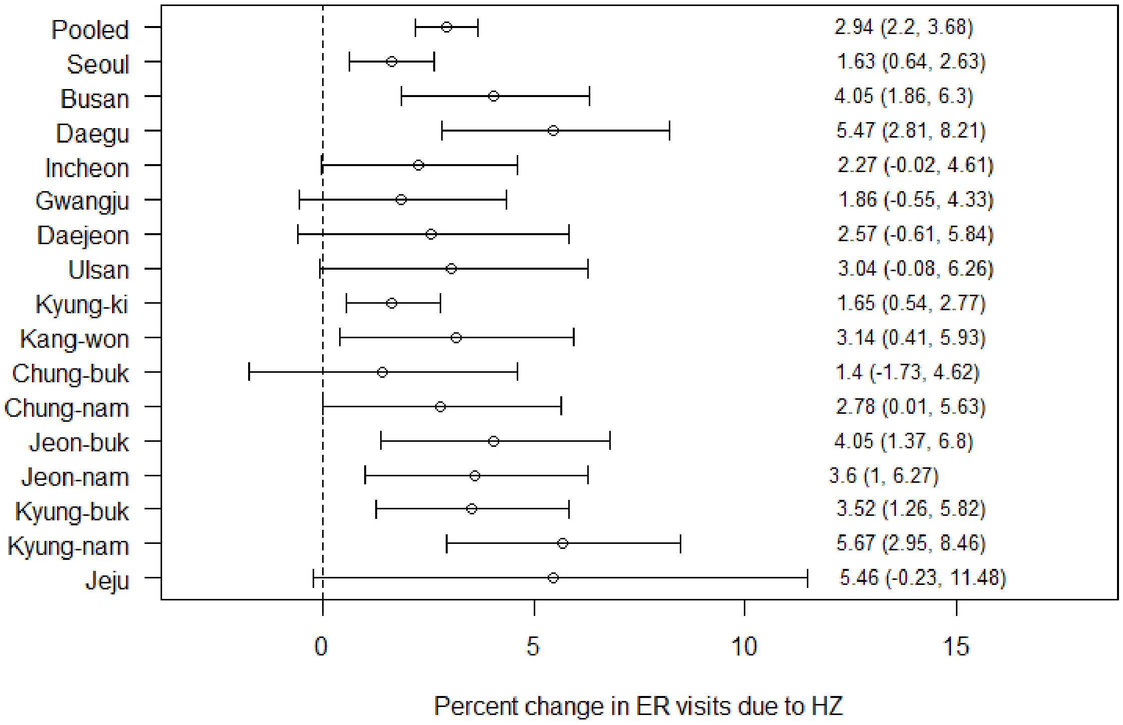

Supplementary Figure 3. Unbiased risk estimator (UBRE) according to degrees of freedom for natural cubic spline function for calendar time at lag 0 for the generalized additive model. Df 8 was chosen, which yielded the lowest UBRE value. (Abbreviation: UBRE, Unbiased risk estimator)

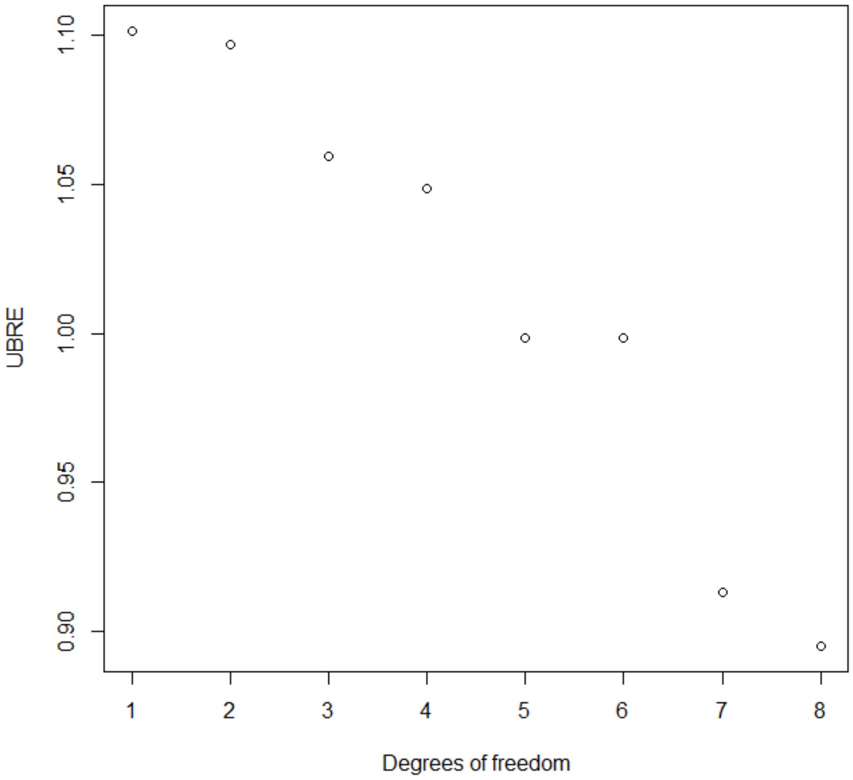

Supplementary Figure 4. Akaike Information Criterion according to degrees of freedom for natural cubic spline function for calendar time at lag 0 for the generalized linear model. Df 8 was chosen, which yielded the lowest AIC value. (Abbreviation: AIC, Akaike Information Criterion)

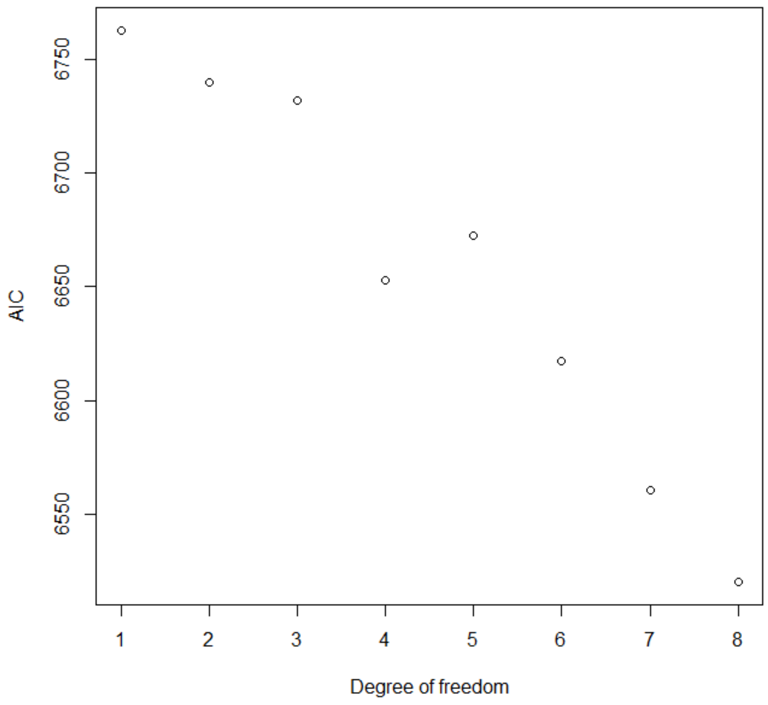

Supplement: Supplementary file 1 — Supplementary information [file 41598_2019_48673_MOESM1_ESM.pdf]
